# Data-driven identification of diagnostically useful extrastriatal signal in dopamine transporter SPECT using explainable AI

**DOI:** 10.1038/s41598-021-02385-x

**Published:** 2021-11-25

**Authors:** Mahmood Nazari, Andreas Kluge, Ivayla Apostolova, Susanne Klutmann, Sharok Kimiaei, Michael Schroeder, Ralph Buchert

**Affiliations:** 1 Department of Computer Science, Biotech, Technical University Dresden, Dresden, Germany; 2 ABX-CRO Advanced Pharmaceutical Services Forschungsgesellschaft M.B.H., 01307 Dresden, Germany; 3 Department of Diagnostic and Interventional Radiology and Nuclear Medicine, University Medical Center Hamburg-Eppendorf, Martinistr. 52, 20246 Hamburg, Germany

**Keywords:** Biomarkers, Neurology

## Abstract

This study used explainable artificial intelligence for data-driven identification of extrastriatal brain regions that can contribute to the interpretation of dopamine transporter SPECT with ^123^I-FP-CIT in parkinsonian syndromes. A total of 1306 ^123^I-FP-CIT-SPECT were included retrospectively. Binary classification as ‘reduced’ or ‘normal’ striatal ^123^I-FP-CIT uptake by an experienced reader served as standard-of-truth. A custom-made 3-dimensional convolutional neural network (CNN) was trained for classification of the SPECT images with 1006 randomly selected images in three different settings: “full image”, “striatum only” (3-dimensional region covering the striata cropped from the full image), “without striatum” (full image with striatal region removed). The remaining 300 SPECT images were used to test the CNN classification performance. Layer-wise relevance propagation (LRP) was used for voxelwise quantification of the relevance for the CNN-based classification in this test set. Overall accuracy of CNN-based classification was 97.0%, 95.7%, and 69.3% in the “full image”, “striatum only”, and “without striatum” setting. Prominent contributions in the LRP-based relevance maps beyond the striatal signal were detected in insula, amygdala, ventromedial prefrontal cortex, thalamus, anterior temporal cortex, superior frontal lobe, and pons, suggesting that ^123^I-FP-CIT uptake in these brain regions provides clinically useful information for the differentiation of neurodegenerative and non-neurodegenerative parkinsonian syndromes.

## Introduction

Neurodegenerative parkinsonian syndromes including Parkinson’s disease (PD) and the rarer atypical neurodegenerative parkinsonian syndromes such as progressive supranuclear palsy (PSP), parkinsonian variant of multiple system atrophy (MSA-P), and corticobasal degeneration are associated with nigrostriatal degeneration resulting in the loss of dopamine transporters (DAT) in the caudate and putamen nuclei of the (dorsal) striatum secondary to the degeneration of pigmented cells in the substantia nigra pars compacta^1,2^. The nigrostriatal degeneration is the major pathophysiological correlate of the motor symptoms in neurodegenerative parkinsonian syndromes. Clinical guidelines recommend single photon emission computed tomography (SPECT) with the DAT ligand *N*-ω-fluoropropyl-2β-carbomethoxy-3β-(4-^123^I-iodophenyl)nortropane (^123^I-FP-CIT) for the detection (or exclusion) of relevant DAT loss in the striatum to support the diagnostic workup in patients with clinically uncertain parkinsonian syndrome (CUPS)^3,4^. In clinical routine, both visual interpretation and semi-quantitative analysis of ^123^I-FP-CIT SPECT are focused on the striatum and its subregions^5–7^.

However, loss of dopaminergic neurons in PD is not restricted to the nigrostriatal pathway. There is also PD-related loss of dopaminergic neurons in the ventral tegmental area that directly project to extrastriatal brain regions including nucleus accumbens, medial prefrontal cortex, hippocampus and amygdala^8–12^. Degeneration of these dopaminergic pathways most likely contributes to cognitive and behavioral symptoms in PD.

As a consequence, the diagnostic accuracy of ^123^I-FP-CIT SPECT might be improved by taking into account extrastriatal signal in addition to the striatal signal. In fact, a previous study provided evidence that taking into account the ^123^I-FP-CIT uptake in the insular cortex might increase the accuracy of ^123^I-FP-CIT SPECT for the detection of PD^13^. This previous study did not find PD-related differences in ^123^I-FP-CIT uptake in the frontal, parietal, and temporal lobes. To some extent this might be explained by limited sensitivity of the a priori defined bilateral regions-of-interest (ROIs) covering the entire brain lobes used in this study. PD-related alterations of extrastriatal ^123^I-FP-CIT uptake may not be uniform throughout entire brain lobes, but they might be restricted to rather small parts within a lobe, for example the orbitofrontal part of the frontal lobe or the amygdala in the temporal lobe^14^. Furthermore, PD-related alterations of extrastriatal ^123^I-FP-CIT uptake might be left–right asymmetric, that is, more pronounced in one hemisphere, similar to PD-related reduction of striatal ^123^I-FP-CIT uptake, which generally is more pronounced in the brain hemisphere contralateral to the side of the body that is more strongly affected by the motor symptoms^15^. Thus, the use of a priori defined ROIs covering the whole bilateral frontal or parietal or temporal lobe might have resulted in considerable ‘dilution’ of more localized and lateralized effects, which in turn reduced the sensitivity to detect them.

Against this background, the aim of the present study was to identify extrastriatal brain regions that might contribute to the differentiation between neurodegenerative and non-neurodegenerative CUPS by ^123^I-FP-CIT SPECT using a deep learning approach based on a custom-made convolutional neural network (CNN)^16,17^ and layer-wise relevance propagation (LRP). This fully data-driven approach does not require a priori hypotheses on which extrastriatal brain regions might provide most information for the differentiation between neurodegenerative and non-neurodegenerative CUPS. Furthermore, this approach is voxel-based and, therefore, is expected to provide high sensitivity for the identification of small and/or lateralized clusters of extrastriatal ^123^I-FP-CIT signal for this task.

The study retrospectively included a large sample of ^123^I-FP-CIT images from clinical routine (n = 1306). The sample was randomly split into training sample, validation sample and test sample in order to improve specificity by reducing the risk of erronously identifying nonrelevant brain regions due to overfitting.

## Results

Overall accuracy, sensitivity, and specificity of the CNN for classification of the ^123^I-FP-CIT SPECT in the test set are given in Table 1, separately for the three settings. The highest accuracy (97.0%) with almost balanced sensitivity and specificity was obtained in the “full image” setting. Overall accuracy in the “striatum only” setting was slightly lower (95.7%), mainly driven by an increased rate of false positive cases (specificity 92.1% versus 96.0% in the “full image” setting). Overall accuracy was strongly reduced (69.3%) in the “without striatum” setting, but still considerably better than chance level (50%). Loss of sensitivity was more pronounced than loss of specificity.

**Table 1:** Overall accuracy, sensitivity, and specificity of the CNN-based classification of the ^123^I-FP-CIT SPECT images in the test set.

Setting	Overall accuracy	Sensitivity	Specificity
“Full image”	97.0	98.0	96.0
“Striatum only”	95.7	99.3	92.1
“Without striatum”	69.3	59.7	78.8

**Figure 1:**
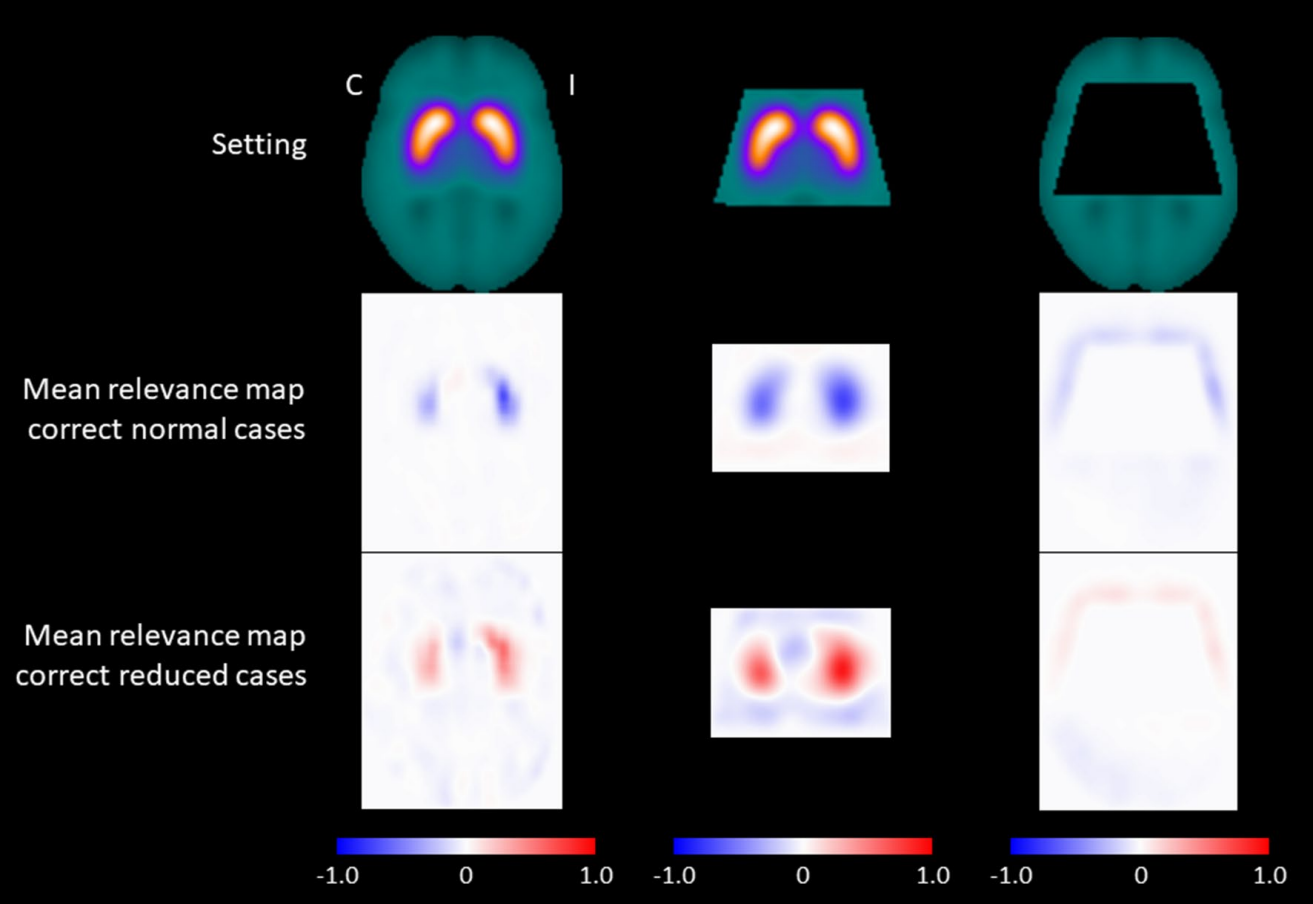
Mean relevance maps. Transversal slice through the striatum of the mean relevance maps of the ^123^I-FP-CIT SPECT images correctly classified as normal (middle row) or correctly classified as reduced (bottom row) by the CNN in the three different settings (“full image”: left column, “striatum only”: middle column, “without striatum”: right column) (*C* contralateral, *I* ipsilateral).

A transversal slice through the striatum of the mean relevance maps of the ^123^I-FP-CIT SPECT images correctly classified by the CNN is shown in Fig. 1, separately for correctly classified normal SPECT and for correctly classified reduced SPECT. The mean relevance map of the correctly classified normal ^123^I-FP-CIT SPECT was the inverse (change of sign) of the mean relevance map of the correctly classified reduced SPECT to good approximation, independent of the setting. This indicates that the same brain regions were the most relevant for classification of the DAT SPECT as normal or reduced, as is to be expected for a binary classification task. This was the rationale for computing a “heat map” by voxel-wise subtraction of the mean relevance map of correctly classified normal ^123^I-FP-CIT SPECT from the mean relevance map of correctly classified reduced ^123^I-FP-CIT SPECT. This was done separately for each of the three settings.

**Figure 2:**
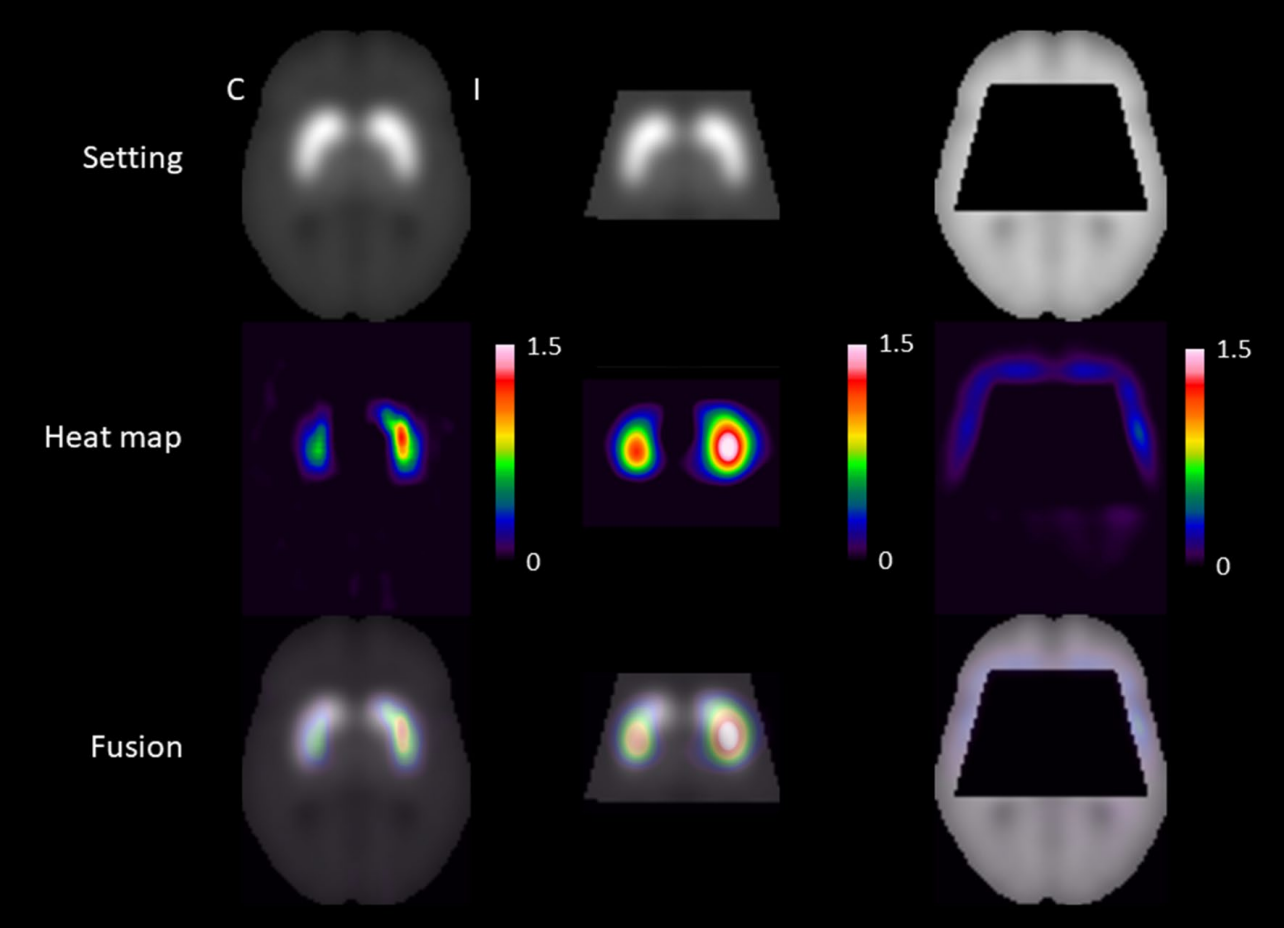
Mean heat maps through the striatum. Transversal slice through the striatum of the mean heat maps (middle row) of the correctly classified ^123^I-FP-CIT SPECT images in the three different settings (“full image”: left column, ”striatum only”: middle column, “without striatum”: right column). The mean heat maps were obtained by voxel-wise subtraction of the mean relevance map of the ^123^I-FP-CIT SPECT images correctly classified as reduced and the mean relevance map of the ^123^I-FP-CIT SPECT images correctly classified as normal by the CNN (Fig. 1) (*C* contralateral, *I* ipsilateral).

**Figure 3:**
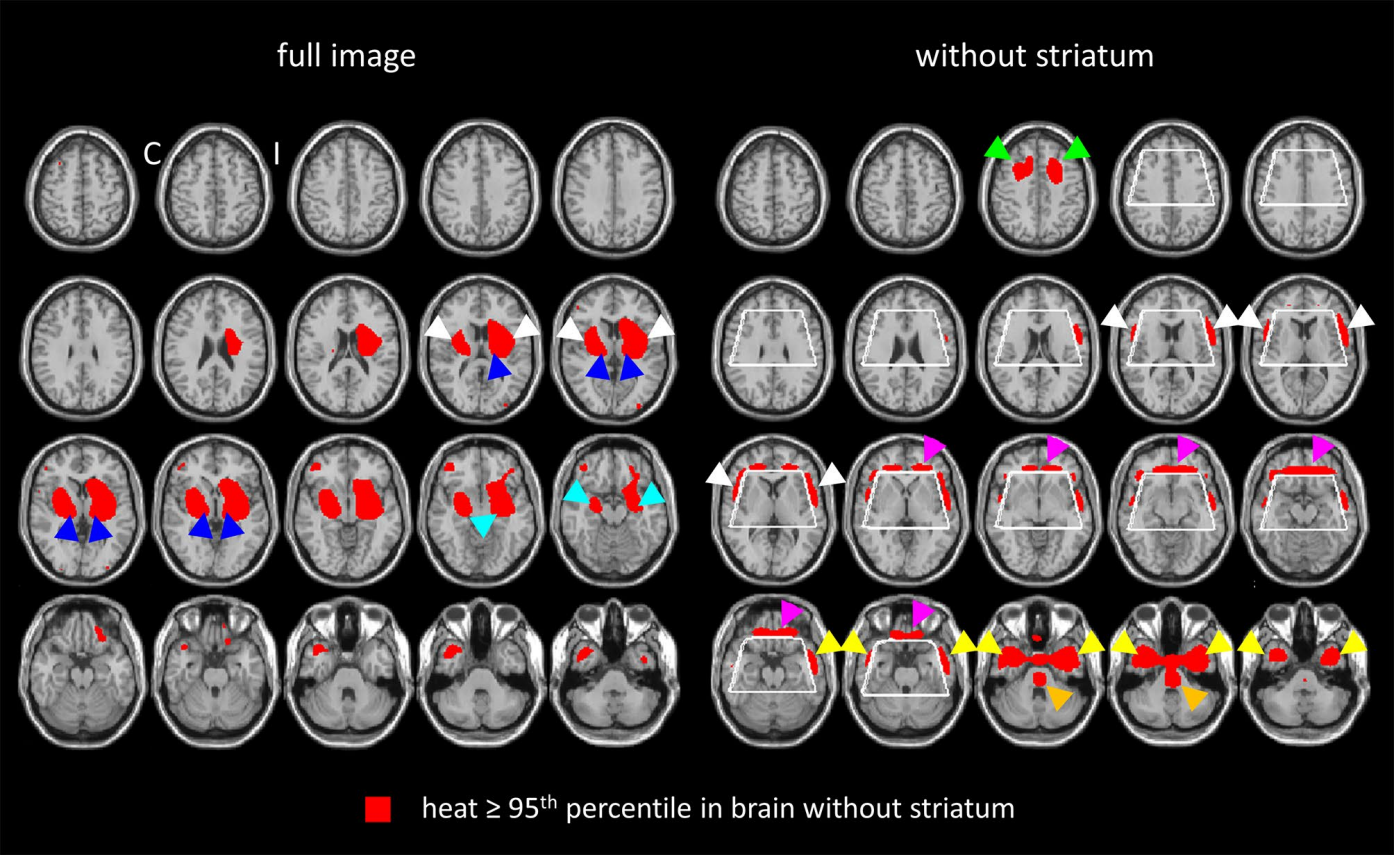
Mean heat maps throughout the whole brain. Mean heat map of the correctly classified ^123^I-FP-CIT SPECT images in the “full image” setting (left) and in the “without striatum” setting (right) overlaid to the single subject T1w-MRI template of SPM12. The mean heat maps were thresholded at the 95th percentile of the heat values in the brain except the striatum region (white contour), separately in both settings. The arrow heads point to the clusters of increased relevance in the insula region (white), the amygdala (aquamarine), the ventromedial prefrontal cortex (purple), the thalamus (blue), the anterior temporal cortex/temporal pole (yellow), the superior frontal lobe (green) and in the pons (orange) (*C* contralateral, *I* ipsilateral).

A transversal slice through the striatum of the resulting heat maps is shown in Fig. 2. Highest relevance was attributed to the ipsilateral putamen, followed by the contralateral putamen and the ipsilateral caudate nucleus in the “full image” setting as well as in the “striatum only” setting. For assessment of extrastriatal relevance, the heat maps of the “full image” setting and of the “without striatum” setting were dichotomized at their 95th percentile and overlaid to the single subject T1w-MRI template of SPM12 (Fig. 3). The relevance clusters in the ipsilateral and in the contralateral striatum in the “full image” setting clearly extended beyond the striatum into the insula region, the thalamus, and into the amygdala region. The relevance cluster in the insula region in both hemispheres was confirmed in the “without striatum” setting, although localization, size and shape of the cluster slightly differed between the “full image” and the “without striatum” setting. At least to some extent this is explained by the fact that parts of the insula region were cut from the brain in the “without striatum” setting (Fig. 3). Thalamus and amygdala were completely cut from the images in the “without striatum” setting and, therefore, could not be assessed in this setting (Fig. 3). Further relevance clusters that were consistently detected in both settings were located in the ventromedial prefrontal cortex and in the anterior temporal cortex/temporal pole in both hemispheres. Additional relevance clusters in the superior frontal lobe and in the pons were detected in the “without striatum” setting only (Fig. 3).

**Figure 4:**
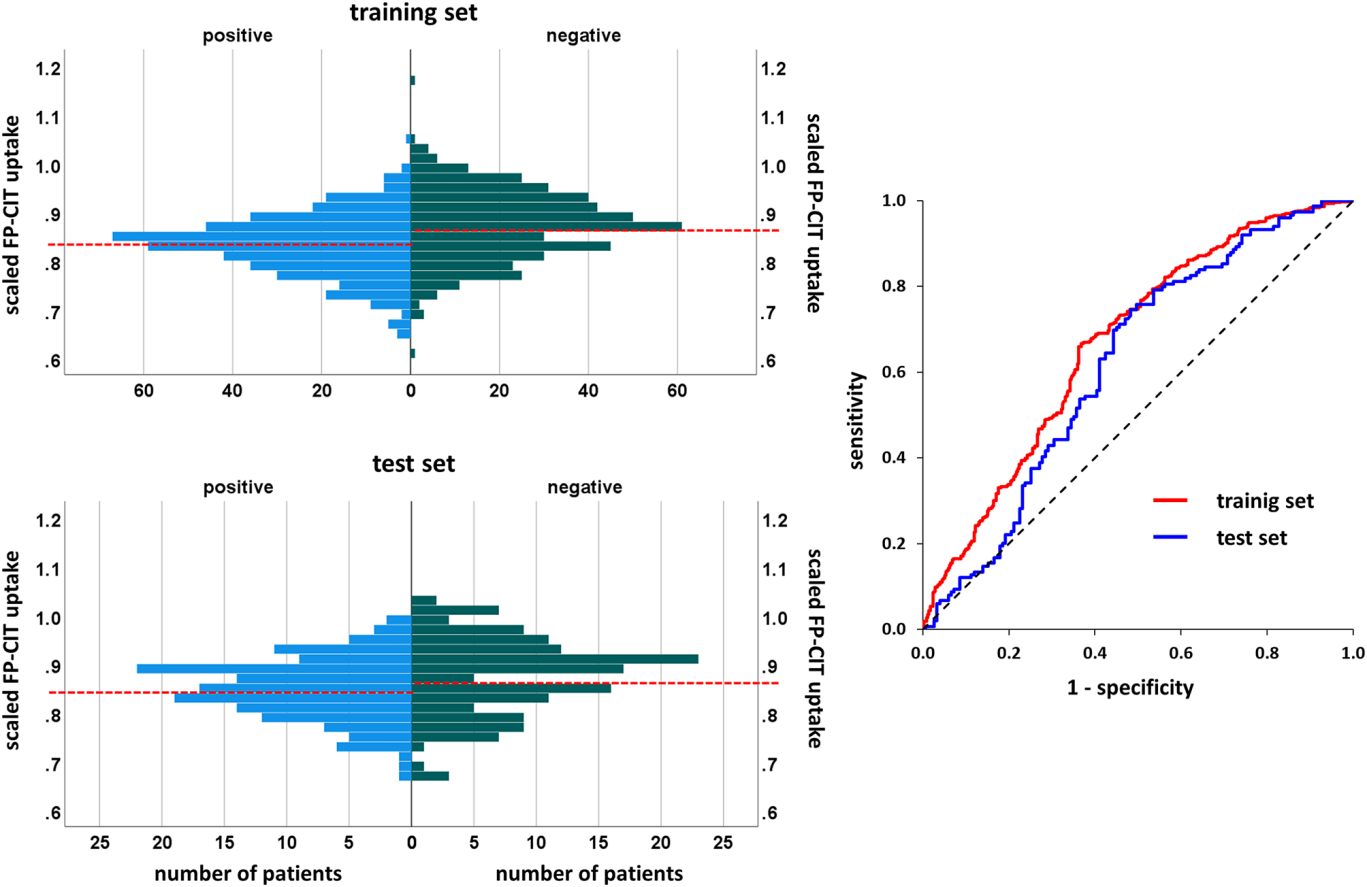
^123^I-FP-CIT uptake in the ipsilateral insula. Histograms and ROC curves of the ^123^I-FP-CIT uptake in the relevance cluster in the ipsilateral insula region (Fig. 3) in the training set and in the test set. The dashed red lines in the histograms indicate their mean values.

In order to further evaluate these findings, the relevance clusters in the “without striatum” setting were used as ROIs to compare the ^123^I-FP-CIT uptake in these clusters between the ^123^I-FP-CIT SPECT images with PD-characteristic reduction of striatal uptake (according to the visual classification) and the ^123^I-FP-CIT SPECT images with normal striatal uptake. In the training set, the ^123^I-FP-CIT uptake was significantly reduced in the ^123^I-FP-CIT SPECT images with reduced striatal uptake in the insula, ventromedial prefrontal cortex, and anterior temporal cortex/temporal pole in both hemispheres. The extrastriatal ^123^I-FP-CIT uptake was not significantly asociated with the striatal status in the superior frontal cortex and in the pons. In the test set, only the reduction of the ^123^I-FP-CIT uptake in the ipsilateral and in the contralateral insula cluster in ^123^I-FP-CIT SPECT images with reduced striatal signal remained statistically significant (P ≤ 0.001). Receiver operating characteristic (ROC) analysis of the ^123^I-FP-CIT uptake in the ipsilateral insula with respect to the differentiation between reduced and normal ^123^I-FP-CIT SPECT revealed an area of 0.668 (95%-confidence interval 0.633–0.704, P < 0.0005) under the ROC curve in the training set and an area of 0.621 (95%-confidence interval 0.557–0.684, P < 0.0005) in the test set (Fig. 4).

**Figure 5:**
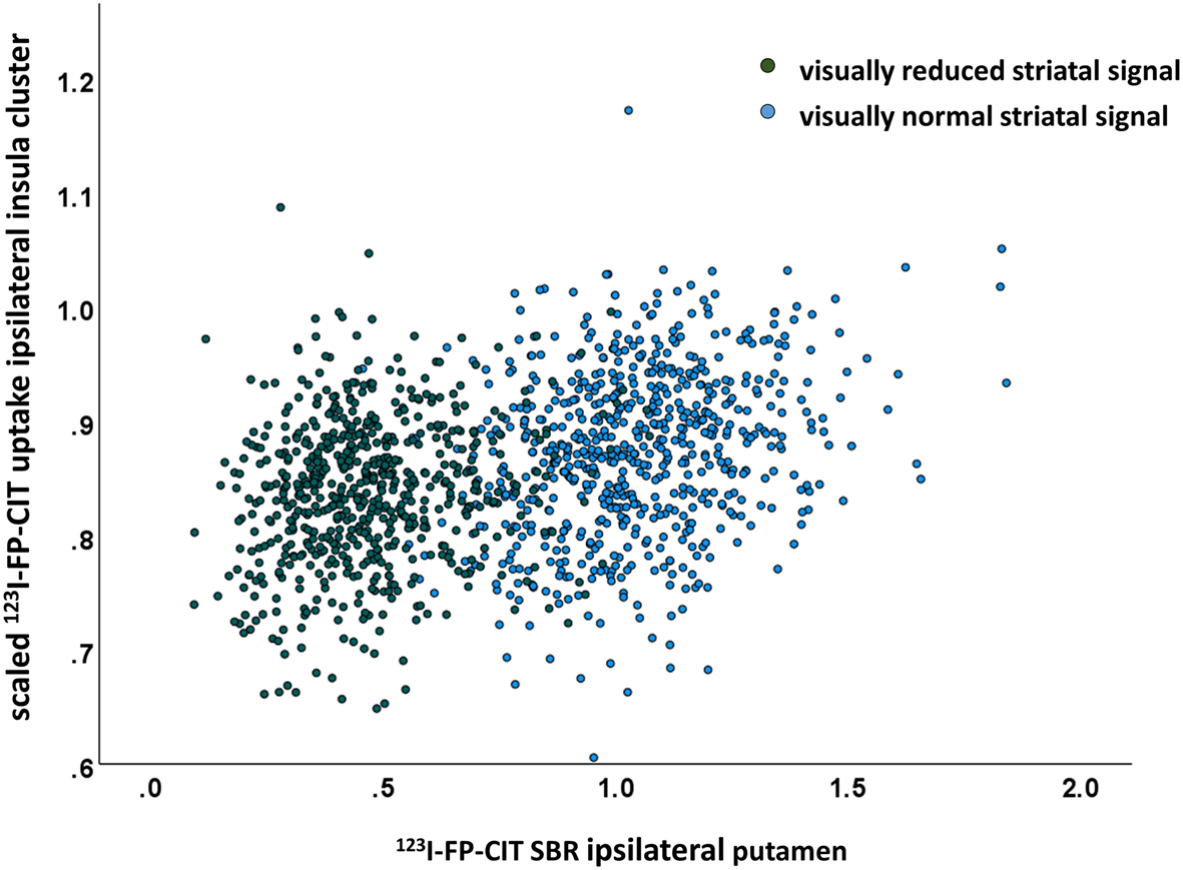
Scatterplot of the scaled ^123^I-FP-CIT uptake in the ipsilateral insula cluster identified by LRP in the “without striatum” setting (Fig. 3) versus the specific binding ratio (SBR) of ^123^I-FP-CIT in the ipsilateral putamen in the whole sample (n = 1306). The color of the symbols indicates PD-typical reduction of (green) or normal (blue) striatal ^123^I-FP-CIT uptake according visual interpretation.

The ^123^I-FP-CIT uptake in the ipsilateral insula cluster was positively correlated with the ^123^I-FP-CIT SBR in the ipsilateral putamen in the whole patient sample (n = 1306, Pearson correlation coefficient R = 0.331, P < 0.0005, Fig. 5). The correlation was also significant in the subset of ^123^I-FP-CIT SPECT images with PD-typical reduction of the striatal signal according to visual inspection (R = 0.158, P < 0.0005) as well as in the subset of ^123^I-FP-CIT SPECT images with normal striatal signal according to visual inspection (R = 0.270, P < 0.0005).

## Discussion

This study provides further evidence of extrastriatal alterations in ^123^I-FP-CIT SPECT with typical striatal reduction that might be clinically useful for the differentiation between neurodegenerative and non-neurodegenerative parkinsonian syndromes. CNN-based automatic classification of ^123^I-FP-CIT SPECT images performed slightly worse in the “striatum only” setting compared to the “full image” setting (overall accuracy in the test set 97.0% versus 95.7%) suggesting that relevant (extrastriatal) information was missing in the “striatum only” setting. This was confirmed by CNN-based classification accuracy in the “without striatum” setting (69.3%) clearly above chance level.

For the identification of the extrastriatal brain regions that most strongly contributed to CNN-based classification of ^123^I-FP-CIT SPECT, layer-wise relevance propagation (LRP) was used. This fully data-driven approach identified the bilateral insula as the most relevant extrastriatal brain region. The ^123^I-FP-CIT uptake in the ipsilateral insula cluster was positively correlated with the ^123^I-FP-CIT SBR in the ipsilateral putamen in the whole sample as well as in the subset of ^123^I-FP-CIT SPECT images with normal striatal signal according to visual inspection. This suggests an association between the loss of putaminal DAT and the loss of insular ^123^I-FP-CIT binding sites in neurodegenerative parkinsonian syndromes as well as a physiological association between the density of dopaminergic innervation of the putamen and the density of monoaminergic innervation of the insula in subjects without nigrostriatal degeneration. Further extrastriatal relevance of ^123^I-FP-CIT uptake was identified by LRP in the amygdala, ventromedial prefrontal cortex, thalamus, anterior temporal cortex/temporal pole, superior frontal lobe, and in the pons.

The diagnostic relevance of the ^123^I-FP-CIT uptake in the amygdala and in the ventromedial prefrontal cortex might be related to the degeneration of further dopaminergic pathways in addition to the nigrostriatal pathway, particularly the mesocortical pathway from the ventral tegmental area to the prefrontal cortex and the mesoamygdaloid pathway from the ventral tegmental area to the amygdala.

However, degeneration of the serotonergic neurotransmitter system^18^ might also have contributed to the observed diagnostic relevance of extrastriatal signal in ^123^I-FP-CIT SPECT, as ^123^I-FP-CIT binds also to the serotonin transporter (SERT)^19^, although with about three times lower affinity than to the DAT^19,20^. This is supported by the finding that ^123^I-FP-CIT binding in SERT-rich brain regions can be blocked by selective serotonin reuptake inhibitors^21,22^, but not by selective DAT blockers^21,23^. Furthermore, extrastriatal ^123^I-FP-CIT uptake declines during healthy aging^24,25^. In some extrastriatal brain regions, including insulo-opercular and the anterior cingulate/medial frontal cortices, thalamus, and pons, the rate of the age-related percentage decline is higher than in the striatum^24,26^. For example, Koch et al.^26^ reported an 8% decline per decade of the specific ^123^I-FP-CIT binding ratio in the thalamus, considerably larger than the 4% per decade striatal decline observed in the same study. This is in good agreement with the age-related decline of 9.6% per decade of the non-displaceable binding potential of the SERT ligand [^11^C](+)McN565 in the thalamus^27^. Binding to the norepinephrine transporter can be neglected in ^123^I-FP-CIT SPECT, because the affinity of ^123^I-FP-CIT for the norepinephrine transporter is about 40 times lower than for the DAT^19^.

The findings of the present study are in good agreement with previous studies. Pilotto et al. compared extrastriatal SBR of ^123^I-FP-CIT between 56 non-demented patients with clinical diagnosis of PD and 54 control patients with clinical diagnosis of isolated action or rest tremor syndrome and visually normal ^123^I-FP-CIT SPECT using a priori defined anatomical ROIs in the frontal, parietal, temporal and cingulate cortices, and in the insula, thalamus and midbrain^13^. Amongst these extrastriatal brain regions, only the insula and the thalamus showed a significant effect (reduced SBR in the PD group). Discriminant analysis demonstrated the ^123^I-FP-CIT SBR in the insula to be the best single extrastriatal parameter for the detection of PD. The authors concluded that “assessment of insular ^123^I-FP-CIT SBR might increase the accuracy of classical nigrostriatal evaluations in PD patients”^13^.

Nicastro et al. performed ROI-based analyses with MRI-based partial volume correction of ^123^I-FP-CIT SPECT in a clicical sample of 157 patients with neurodegenerative parkisonian syndrome comprising PD, MSA-P, PSP, corticobasal syndrome (CBS), and dementia with Lewy bodies (DLB) together with 58 control subjects with parkinsonism or tremor not asociated with dopaminergic degeneration^28^. The proportion of patients with an atypical neurodegenerative parkisonian syndrome (MSA-P, PSP, CBS, DLB) amongst the patients with nigrostriatal degeneration was considerably higher than in the present study (62% compared to about 10%). Statistical testing with correction for age, sex and the use of antidepressant medication (selective serotonin reuptake inhibitors, SSRI/serotonin and norepinephrine reuptake inhibitors, SNRI) revealed a significant reduction of the specific ^123^I-FP-CIT binding ratio in caudate nucleus, putamen, pallidum and insula in each diagnostic subgroup of the patients with neurodegenerative parkinsonian syndrome. In addition, the specific ^123^I-FP-CIT binding ratio was reduced in the thalamus in PSP and MSA-P patients, in the midbrain in PD and PSP patients, and in the amygdala in PSP patients^28^. ROC analyses demonstrated a significant improvement in the differentiation of the whole group of patients with neurodegenerative parkinsonian syndrome from the controls when including the extrastriatal signals in the model^28^.

Premi and co-workers, using independent component analysis of the whole brain, identified six spatial covariance patterns in ^123^I-FP-CIT SPECT images of 84 PD patients and a control group of 59 patients with a tremor syndrome without nigrostriatal degeneration^29^. The covariance patterns identified by the multivariate analysis included cortical, thalamic and brain stem regions in addition to the striatum despite the fact that the reduction of ^123^I-FP-CIT binding in the PD patients revealed by conventional univariate voxel-based testing was restricted to the bilateral striatum^29^.

Ouchi and co-workers performed positron emission tomography (PET) with the DAT ligand ^11^C-beta-CFT in eight unmedicated early stage PD patients and six healthy control subjects^14^. Using tracer kinetic modelling of time activity curves from dynamic PET imaging and the input function generated from arterial blood samples, these authors estimated the ^11^C-beta-CFT binding potential in the orbitofrontal cortex and in the amygdala. The ^11^C-beta-CFT binding potential was significantly reduced in the PD patients in both regions. The authors concluded that orbitofrontal and amygdalar presynaptic dopaminergic functions are reduced in early PD and that this might be a pathophysiological correlate of cognitive and behavioral alterations in PD^14^. Given that (1) ^11^C-beta-CFT is particularly selective to the DAT (compared to other monoamine transporters)^30^ and (2) dopaminergic axon terminals have been found in the orbitofrontal cortex and the amygdala^31^, Ouchi and co-workers assumed that the reduction of the ^11^C-beta-CFT binding potential observed in their study indicates loss of dopaminergic axon terminals in the orbitofrontal cortex and the amygdala in PD.

Oh and co-workers, combining resting-state functional MRI and DAT-PET with ^18^F-FP-CIT in 59 patients with clinically diagnosed PD, reported altered intrinsic functional activity of the right insular cortex that was correlated with decreased DAT availability in the caudate nucleus as well as with lower performance in executive, visuospatial and language tasks^32^.

Nocker et al.^33^ and Joling et al.^34^ reported that extrastriatal signals in DAT-SPECT might also contribute to the differentiation of atypical neurodegenerative parkinsonian syndromes from PD, particularly PSP and MSA-P. Alterations of extrastriatal signal in DAT-SPECT might also contribute to a better understanding of the pathophysiological mechanisms underlying psychiatric symptoms^35–37^ or altered pain perception^38^ in PD.

The clinical utility of extrastriatal ^123^I-FP-CIT signals might be limited by somewhat lower test–retest stability compared to the striatal signal (3.6–9.1% test–retest variability in the lateral frontal/temporal cortex and combined cortical regions^39^). Reduced test–retest stability of extrastriatal signals in clinical ^123^I-FP-CIT SPECT might be related to the fact that the optimal time frame for imaging SERT with ^123^I-FP-CIT is between 2 and 3 h post intravenous injection, which is somewhat earlier than the 3–6 h time window for DAT imaging^40^.

The following limitations of this study should be noted. First, patients had not been asked to discontinue antidepressant medication with SSRI or SNRI, because SSRI and SNRI do not significantly affect visual interpretation of ^123^I-FP-CIT SPECT^41^. However, small (about 10%) increases of the striatal ^123^I-FP-CIT SBR under SSRI/SNRI medication have been reported, presumably due to the blocking of ^123^I-FP-CIT binding to SERT in the (extrastriatal) reference region used to estimate the non-displaceable binding of ^123^I-FP-CIT^41^. It cannot be ruled out, therefore, that the findings of the present study were affected by blocking of extrastriatal SERT by SSRI/SNRI medication, particularly if SSRI/SNRI usage differed between patients with versus patients without nigrostriatal degeneration. This, however, might not be expected. A recent retrospective study including a similar sample of patients from clinical routine found no difference between patients with neurodegenerative parkinsonian syndrome and patients with non-neurodegenerative parkinsonian syndrome with respect to the proportion of patients under SSRI/SNRI treatment^28^. In the present study, information about SSRI/SNRI use was not available in the vast majority of the patients. Most sites do not ask patients to discontinue SSRI/SNRI prior to ^123^I-FP-CIT SPECT in clinical routine so that the findings of the present study might be translated to everyday clinical routine at most sites. Second, ^123^I-FP-CIT SPECT images were not corrected for photon attenuation in this study. The rationale for this was that neither visual interpretation nor semi-quantitative analysis of ^123^I-FP-CIT SPECT necessarily benefit from correction of attenuation (and/or scatter and/or septal penetration), although values of the ^123^I-FP-CIT SBR depend on whether and how attenuation correction is performed^5,42^. As a consequence, many sites do not perfom attenuation correction in ^123^I-FP-CIT SPECT in clinical routine, not only to save the radiation dose to the patient in CT-based attenuation correction or the technician’s time for manual or semi-automatic delineation of the outer contour of the head for Chang attenuation correction, but also to avoid artifacts by the attenuation correction that might affect visual interpretation (e.g., apparent left–right asymmetry of the striatal signal caused by head motion between the low-dose CT and the SPECT acquisition, or by inaccurate delineation of the outer contour of the head by less experienced technicians). However, correct attenuation correction might reduce between-subjects variability of no interest (associated with varying head size) in ^123^I-FP-CIT SPECT and, therefore, might improve the power for the detection of clinically useful extrastriatal signal. Finally, visual interpretation as reduced or normal striatal ^123^I-FP-CIT uptake by an experienced reader was used as standard-of-truth in this study. The clinical diagnosis after a follow-up of ≥ 12 months would have been prefered as standard-of-truth but was available in less than 10% of the included patients. Amongst the patients with neurodegenerative parkinsonian syndrome included in this study most likely about 10% suffered from PSP, MSA-P, CBS or DLB rather than PD, which might have affected the findings. For example, patients with PSP or MSA-P might have contributed to the increased pontine relevance for the classification of ^123^I-FP-CIT SPECT images^43^.

In conclusion, the present study provides further evidence that alterations of ^123^I-FP-CIT uptake in extrastriatal brain regions including insula, amygdala, ventromedial prefrontal cortex, thalamus, anterior temporal cortex/temporal pole, superior frontal lobe, and pons might be used to improve the accuracy of clinical ^123^I-FP-CIT SPECT for the differentiation of neurodegenerative and non-neurodegenerative parkinsonian syndromes.

## Methods

### ^123^I-FP-CIT SPECT data

The PACS of the Department of Nuclear Medicine of the University Medical Center Hamburg Eppendorf was searched using the following inclusion criteria: (I1) ^123^I-FP-CIT SPECT had been performed in clinical routine to support the etiological diagnosis of a CUPS, (I2) ^123^I-FP-CIT SPECT had been performed with a double head SPECT system equipped with low-energy-high-resolution parallel-hole collimators according to standard procedure guidelines^44^, and (I3) raw projection data were digitally available for consistent retrospective image reconstruction. No exclusion criteria were applied. This resulted in the inclusion of 1306 ^123^I-FP-CIT SPECT. Mean age of the included patients was 67.5 ± 11.2 years (range 20–90 years), 41.8% of the patients were females. The activity dose of ^123^I-FP-CIT injected intravenously ranged between 139 and 199 MBq (mean 184 ± 10 MBq). Patients had discontinued medication and drugs of abuse that may significantly interfere with the visual interpretation of ^123^I-FP-CIT SPECT (cocaine, amphetamine, metamphetamine, dextroamphetamine, methylphenidat, modafinil, amfepramone, mazindol, phentermine or ephedrines, bupropion, radafaxine, fentanyl, ketamine, isoflurane, and phencyclidine)^5,44^. Patients had not discontinued selective serotonin reuptake inhibitors (SSRI) nor serotonin and norepinephrine reuptake inhibitors (SNRI) that do not significantly affect visual interpretation of ^123^I-FP-CIT SPECT^41^.

The projection data were reconstructed to tomographic SPECT images using filtered backprojection and a Shepp-Logan filter with cutoff 1.25 cycles/cm^45^. Neither attenuation correction nor scatter correction were applied^46^. Image reconstruction was performed using the “iradon” function of MATLAB (www.mathworks.com). All 1306 projection data were reconstructed fully automatically in a single batch using a custom MATLAB script in order to avoid errors by manual interaction.

Individual SPECT images were transformed (affine) into the anatomical space of the Montreal Neurological Institute (MNI) using the Statistical Parametric Mapping software package (version SPM12)^47^ and a custom-made ^123^I-FP-CIT template. Voxel intensities were scaled to the 75^th^ percentile in a reference region comprising whole brain except striata, thalamus, brain stem, and ventricles^26,48^.

The ^123^I-FP-CIT SPECT images were classified as ‘reduced’ (PD-characteristic reduction of striatal ^123^I-FP-CIT uptake) or ‘normal’ by an experienced reader based on visual inspection of a standardized display of the stereotactically normalized SPECT images^49^. The reader was blinded for all clinical information. Binary classification of the images was repeated by the same reader in a second reading session. Images with discrepant classification in the two reading sessions (29 of the 1306 cases, 2.2%) were assessed a third time by the same reader to obtain an “intra-reader consensus” that then was used as standard-of-truth in the further analyses (reduced: n = 637, 48.8%, normal: n = 669, 51.2%).

Clinical follow-up was not available in the vast majority of the included patients. From the subsample of patients in whom clinical follow-up was available it might be assumed that amongst the patients with reduced ^123^I-FP-CIT SPECT about 90% suffered from PD (without and with cognitive impairment) whereas the remaining 10% had an atypical neurodegenerative parkinsonian syndrome including parkinsonian variant of multiple system atrophy (MSA-P), progressive supranuclear palsy (PSP), corticobasal syndrome (CBS), and dementia with Lewy bodies (DLB)^50^. The diagnoses of the patients with normal ^123^I-FP-CIT SPECT most likely included essential tremor, drug-induced parkinsonism, various types of dystonia, psychogenic parkinsonism, and various other diagnoses not associated with nigrostriatal degeneration^50^. The patient sample is representative of everyday clinical routine at the Department of Nuclear Medicine of the University Medical Center Hamburg-Eppendorf.

### Image preprocessing for automatic classification

Specific ^123^I-FP-CIT binding to the DAT in the unilateral putamen was characterized by the specific binding ratio (SBR) of ^123^I-FP-CIT estimated by hottest voxels analysis as described previously^50^, separately in both hemispheres. Stereotactically normalized ^123^I-FP-CIT SPECT images in which the putaminal SBR was lower in the right hemisphere were left–right mirrored at the midsagittal plane such that the putaminal SBR was lower in the left hemisphere in all cases. In the following, the left and right hemisphere are referred to as ‘ipsilateral’ and ‘contralateral’ hemisphere, respectively.

**Figure 6:**
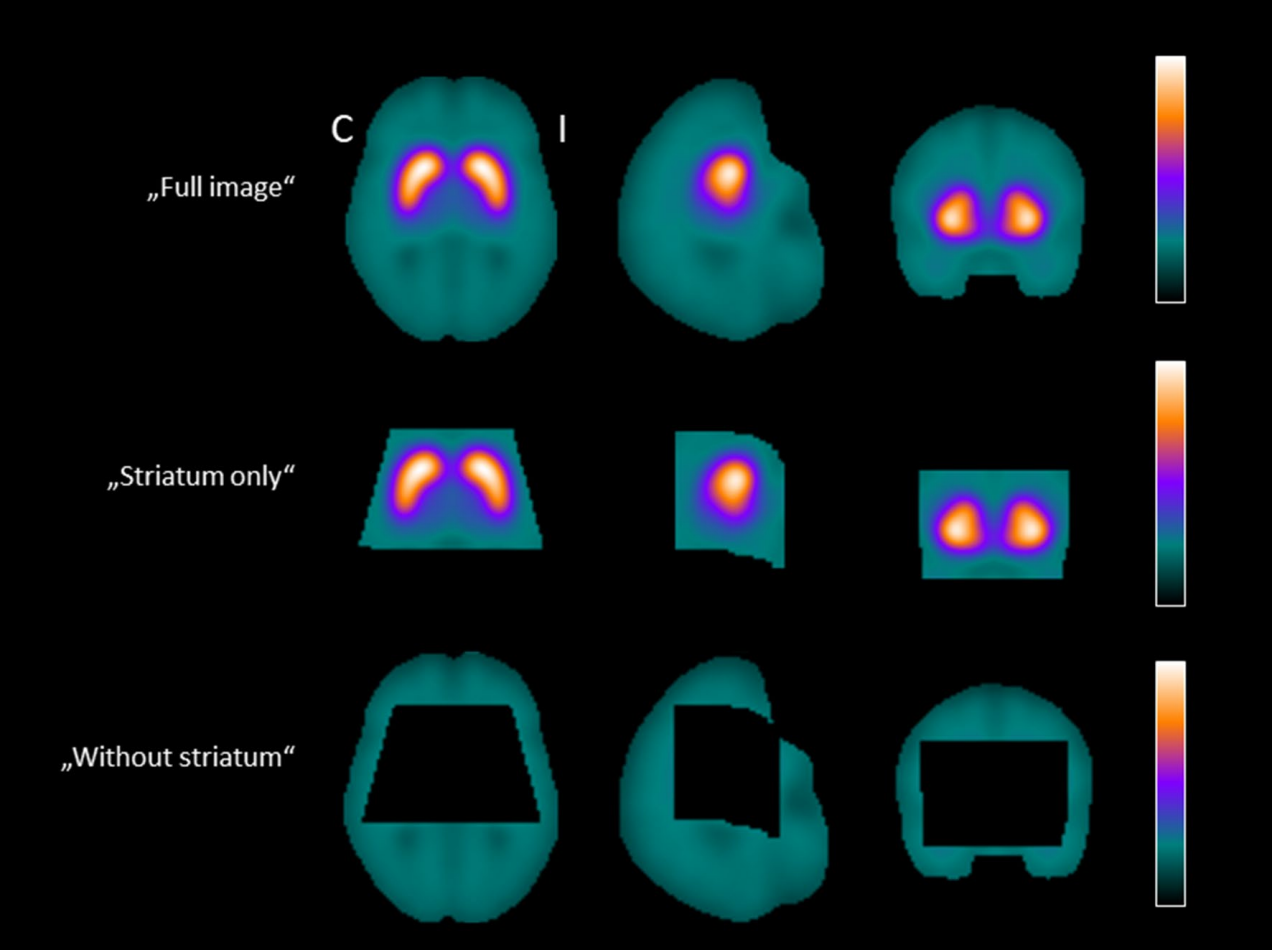
Settings for the CNN training: (i) full 3-dimensional ^123^I-FP-CIT SPECT images (“full image”), (ii) the 3-dimensional region of the striata cropped from the full image (“striatum only”), and (iii) full 3-dimensional ^123^I-FP-CIT SPECT with the region of the striata removed (“without striatum”). The mask of the striatal region used to generate the images for the “striatum only” and the “without striatum” settings was chosen with large safety margin around the striata in order to largely eliminate spill-out of striatal signal into the rest of the brain (*C* contralateral, *I* ipsilateral).

Three different settings were tested for automatic classification of ^123^I-FP-CIT SPECT (Fig. 6). In the “full image” setting, the CNN was trained for classification of the complete 3-dimensional SPECT image (71 × 90 × 72 voxels of 2 × 2 × 2 mm^3^). In the “striatum only” setting, a 3-dimensional image (61 × 44 × 35 voxels) covering the whole striatum in both hemispheres was cropped from the full 3-dimensional ^123^I-FP-CIT SPECT and then used for the CNN training. The full ^123^I-FP-CIT SPECT images with the same 3-dimensional striatum region removed were used for the CNN training in the “without striatum” setting (71 × 90 × 72 voxels). The 3-dimensional striatum region was chosen big enough to largely eliminate spill-out (by partial volume effects) of striatal signal into the rest of the brain that might contaminate the “without striatum” setting by striatal signal.

### Convolutional neural networks

**Figure 7:**
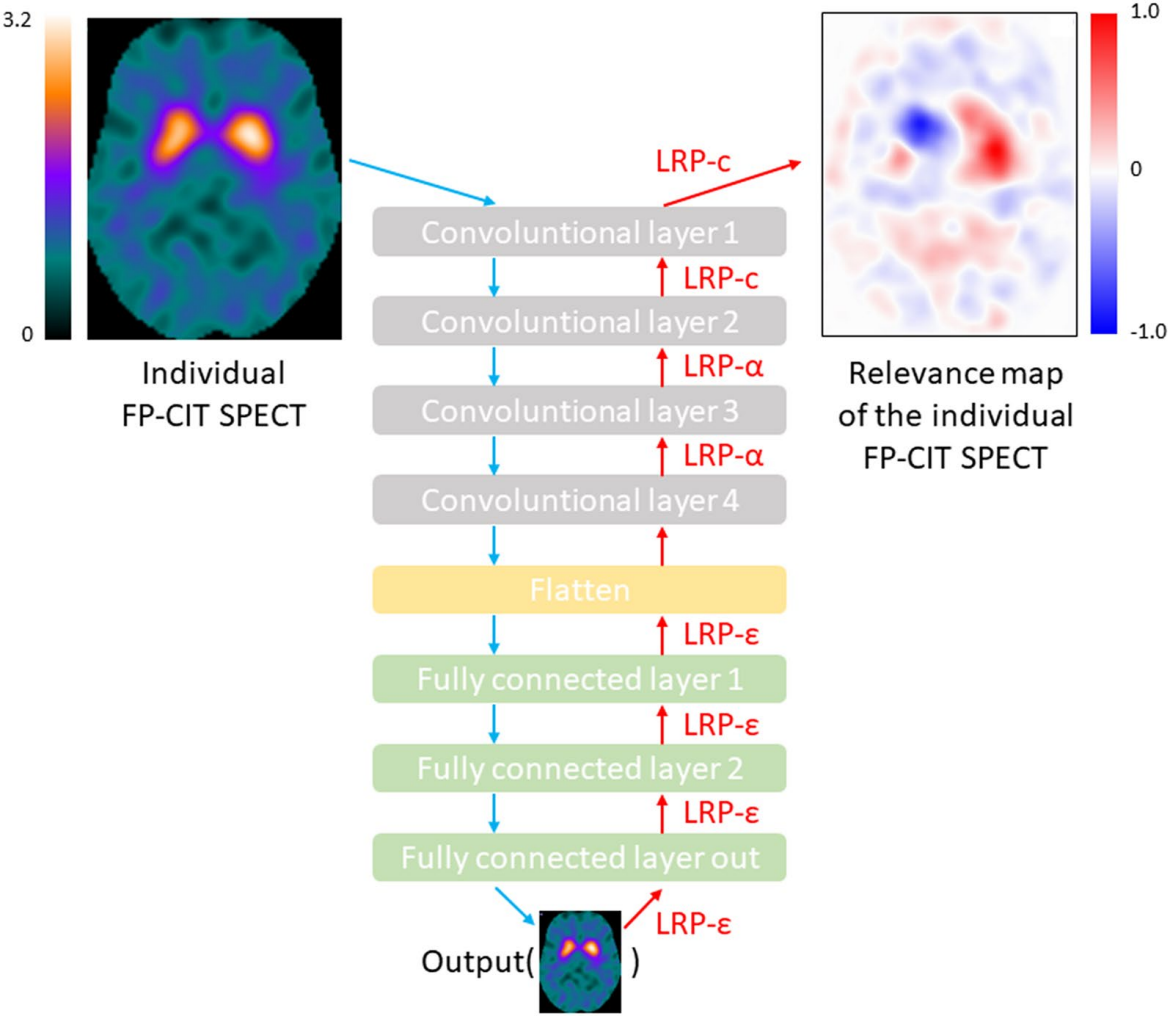
Structure of the CNN used for binary classification of the ^123^I-FP-CIT SPECT images. The same CNN structure was used for each of the three setting (full image, striatum only, without striatum). The CNN was trained separately for each setting resulting in three different CNN. The LRP redistribution rules at the different CNN layers are shown in red.

The structure of the custom-made CNN trained for automatic classification of ^123^I-FP-CIT SPECT is shown in Fig. 7. The same structure was used for each of the three different settings. The CNN comprised four 3-dimensional convolutional layers with 16 filters, kernel size of 3 × 3 × 3. Stride and dilation were set to 1. The convolutional layers were followed by three fully connected neuron layers each with 16 neurons, followed by a 2-way softmax output layer for binary classification. The rectified linear unit was used as activation function at all hidden layers. No pooling layers were used, mainly because all input images were in MNI space so that translation invariance was not required, but also to achieve a simple form of routing which routes all the features in the lower layer to the higher layer^51^. Drop out (0.2) was implemented in the first fully connected layer only. The total number of trainable CNN parameters was 236 million for the ”full image” and the “without striatum” settings, it was 25 million for the striatum only setting.

For the training of the CNN, the subjects were randomly split into training sample (n = 876: 453 normal, 423 reduced), validation sample (n = 130: 65 normal, 65 reduced) and test sample (n = 300: 151 normal, 149 reduced). All subsamples of the random split were well balanced with respect to age and sex. Univariate analysis of variance of age or sex as dependent variable and subset (training, validation, test) and visual classification of the DAT SPECT (normal, reduced) as fixed factors did not reveal any significant effect, despite the rather large sample size providing sufficient statistical power to detect also rather small differences (age: P = 0.592, 0.071, 0.373 for subset, visual classification and interaction of subset × visual classification; sex: P = 0.415, 0.694, 0.288 for subset, visual classification and interaction of subset × visual classification). The same random split was used for all settings. The validation dataset was used only to check for overfitting during the training (no model selection).

The CNN was trained with a batch-size of 8 against the categorical cross-entropy loss using the Adam optimizer with learning rate of 10^–4^. Loss weighting for different classes was not used, because the data were balanced with respect to the class to good approximation.

Using a Nvidia Titan XP graphic card with 12 GB graphic memory, the training of the CNN for “full image” and “without striatum” settings took approximately 72 s per epoch. The CNN could be trained without noticeable overfitting and converged in less than 50 epochs in the “full image” setting. The total training time until convergence was approximately one hour. In the “without striatum” setting, the CNN was trained for 200 epochs and the total training time until convergence was approximately 4 h. In the “striatum only” setting, the training of the CNN took 16 s per epoch. The CNN could be trained without noticeable overfitting and converged in less than 50 epochs with total training time until convergence of approximately 15 min.

### Layer-wise relevanvce propagation

CNN-based classification of medical images is often considered a black-box approach, because it is difficult to retrospectively identify the features learned during the training^52^. Layer-wise relevance propagation (LRP) is an explainable AI technique that allows generation of an individual relevance map for each individual image^53^. The individual relevance map is in the same space as the input image and its voxel intensity values indicate the relevance/importance of the voxels for the CNN-based classification of this image^54^.

In order to estimate the relevance of each single voxel of the subject’s image for the classification of the whole image by the CNN, LRP takes advantage of the CNN graph structure for layer-wise redistribution of relevance from the most activated output neuron up to the input layer^53,55^. More precisely, LRP is based on a local redistribution rule to redistribute relevance from neurons in a given layer to the neurons in the preceding layer. If *z*_*ij*_ denotes the fraction of the relevance ${R}_{j}^{\left[k\right]}$ at neuron *j* in the CNN layer *k* that is redistributed to neuron *i* in the preceding layer *k-1*, then the total relevance ${R}_{i}^{\left[k-1\right]}$ at neuron *i* is given by

1 $${R}_{i}^{[k-1]}=\sum_{j\in [k]}\frac{{z}_{ij}}{{\sum }_{i\in [k-1]}{z}_{ij}}{R}_{j}^{[k]}$$

The scaling factors $\sum\nolimits_{i\in [k-1]}{z}_{ij}$ in the denominator on the right-hand side guarantee that the relevance is preserved during redistribution at each neuron. When the rectified linear unit is used as activation function, first order Taylor expansion at the prediction point suggests the following standard choice for the redistribution coefficients^56^

2 $${z}_{ij}={a}_{i}{w}_{ij}$$

where ${a}_{i}$ is the activation of neuron *i* for the considered image in the prediction phase (forward pass) and ${w}_{ij}$ is the weight factor for the input to neuron *j* from neuron *i* fixed during the training phase.

Several variations of the LRP rule according to Eqs. (1, 2) have been proposed^56,57^. In the present study three of these variations were combined for (1) improved robustness of LRP by avoiding noise amplification due to the gradient shattering effect^58,59^, (2) reduced spill-out of relevance, and (3) discrimination between features that support the prediction and features that oppose it.

The redistribution rule

3 $$\mathrm{LRP}-\upvarepsilon :{R}_{i}^{[k-1]}=\sum_{j\in [k]}\frac{{z}_{ij}}{{\sum }_{i\in [k-1]}\left\{{z}_{ij}+\epsilon sign\left({z}_{ij}\right)\right\}}{R}_{j}^{[k]}$$

with *z*_*ij*_ according to Eq. (2) was used for relevance redistribution at the fully connected layers close to the output of the CNN (Fig. 7). Here *sign(x)* denotes the sign of x, that is, *sign(x)* = *1* for *x* ≥ *0* and *sign(x)* = *-1* for *x* < *0*. The ε-term is introduced to limit noise amplification. ε = 0.0001 was used.

The redistribution rule

4 $$\mathrm{LRP}-\mathrm{\alpha }:{R}_{i}^{[k-1]}=\sum_{j\in [k]}(\alpha \frac{{z}_{ij}^{+}}{{\sum }_{i\in [k-1]}{z}_{ij}^{+}}+\left(\alpha -1\right)\frac{{z}_{ij}^{-}}{{\sum }_{i\in [k-1]}{z}_{ij}^{-}})$$

with *z*_*ij*_ according to Eq. (2) was used for relevance redistribution at the fourth and the third convolutional layer (Fig. 7). Here “+ ” and “−” indicate the positive and the negative part, respectively, that is

5a $${z}_{ij}^{+}=max(0,{z}_{ij})$$

5b $${z}_{ij}^{-}=min(0,{z}_{ij})$$

The parameter α was chosen as *α* = *2* in order to allow for both positive and negative relevance. Positive relevance indicates that the feature supports the classification decision whereas negative relevance indicates that the feature provides evidence against it.

Finally, uniform redistribution (LRP-c) defined by Eq. (1) with *z*_*ij*_ = *1* was used at the first two layers close to the input of the CNN for improved control of resolution and semantics in the relevance maps^60^ (Fig. 7).

### Statistical analysis

The classification performance of the three different CNN (one for each setting) was estimated in the test set (independent of the training set). Overall accuracy, sensitivity and specificity were used to characterize classification performance.

Mean relevance maps for correctly classified (by the CNN) normal ^123^I-FP-CIT SPECT and mean relevance maps for correctly classified reduced ^123^I-FP-CIT SPECT were obtained by voxel-wise averaging the individual relevance maps of correctly classified normal cases and correctly classified reduced cases, respectively. This was done separately for each setting.

### Ethics declarations

Waiver of informed consent for the retrospective analysis of the clinical data was obtained from the ethics review board of the general medical council of the state of Hamburg, Germany. All procedures performed in this study were in accordance with the ethical standards of the ethics review board of the general medical council of the state of Hamburg, Germany, and with the 1964 Helsinki declaration and its later amendments.

## Data Availability

The datasets generated during and/or analysed during the current study are available from the corresponding author on reasonable request.

## Abbreviations

2d: 2-Dimensional
3d: 3-Dimensional
CBS: Corticobasal syndrome
CUPS: Clinically uncertain parkinsonian syndrome
DAT: Dopamine transporter
DLB: Dementia with Lewy bodies
^123^I-FP-CIT: *N*-ω-Fluoropropyl-2β-carbomethoxy-3β-(4-I-123-iodophenyl)nortropane
LRP: Layer-wise relevance propagation
MSA-P: Parkinsonian variant of multiple system atrophy
PD: Parkinson’s disease
PET: Positron emission tomography
PSP: Progressive supranuclear palsy
ROC: Receiver operating characteristic
ROI: Region-of-interest
SBR: Specific binding ratio
SERT: Serotonin transporter
SNRI: Serotonin and norepinephrine reuptake inhibitor
SPECT: Single-photon emission computed tomography
SSRI: Selective serotonin reuptake inhibitor
